# Downward hyperinflation of the native lung after right single lung transplantation for COPD: A case report highlighting diaphragmatic mobility

**DOI:** 10.1016/j.jhlto.2025.100268

**Published:** 2025-04-08

**Authors:** Shin Tanaka, Tsuyoshi Ryuko, Yasuaki Tomioka, Kazuhiko Shien, Ken Suzawa, Kentaroh Miyoshi, Mikio Okazaki, Seiichiro Sugimoto, Shinichi Toyooka

**Affiliations:** a Department of General Thoracic and Breast and Endocrinological Surgery, Okayama University Graduate School of Medicine, Dentistry and Pharmaceutical Sciences, Okayama, Japan; b Department of General Thoracic Surgery and Organ Transplant Center, Okayama University Hospital, Okayama, Japan

**Keywords:** Chronic obstructive pulmonary disease, Single lung transplantation, Native lung hyperinflation, Diaphragm mobility

## Abstract

A 60-year-old male with COPD underwent right single lung transplantation. Despite progressive hyperinflation of the native left lung, the transplanted lung maintained function, as downward expansion of the left lung displaced the diaphragm without compressing the mediastinum. This suggests diaphragm mobility, aided by the absence of the liver beneath the left diaphragm, contributes to favorable outcomes in right single lung transplantation by preventing mechanical compression of the transplanted lung.

A 60-year-old male with advanced chronic obstructive pulmonary disease (COPD) ( Figure 1a, b) underwent right single lung transplantation. His height and weight were 161 cm and 67 kg, respectively, corresponding to a body mass index (BMI) of 25.8 kg/m². Based on Japanese reference equations,^1^ his predicted forced vital capacity (FVC) was calculated to be approximately 3.31 L. The donor’s predicted FVC, estimated from age and body size, was approximately 4.20 L, resulting in a donor-to-recipient pFVC ratio of 1.27. Accordingly, the donor lungs were approximately 27% larger than the recipient’s predicted lung volume, which is considered acceptable in transplantation for COPD. His postoperative recovery was uneventful, with stable respiratory function and preserved activity of daily living (ADL) (Figure 1c, d). In the second postoperative year, progressive hyperinflation of the native left lung was observed (Figure 1e, g). Lung volume measurements during maximum inspiration showed an increase in left lung volume from 3174 mL to 3502 mL, representing a 328 mL expansion, or over 10% growth. Despite concerns about potential compression of the transplanted right lung, no significant reduction in right lung volume was observed. However, a modest reduction in right lung volume was noted between the second and fourth postoperative years (from 1335 mL to 1228 mL), which may reflect mild compression by the expanding native lung. Although this did not translate into clinical deterioration in our patient, it suggests that some degree of graft compression may have occurred. Given inter-individual variation in mediastinal compliance and diaphragm excursion, the extent of such compression and its physiological impact likely differ among patients. The right lung volume initially increased from 1268 mL to 1335 mL before stabilizing at 1228 mL. Serial chest radiographs indicated that the hyperinflated native left lung expanded downward, pressing on the diaphragm rather than the mediastinum (Figure 1d, f, h). The patient experienced no respiratory decline, and his ADL remained unchanged (Figure 2).

**Figure 1:**
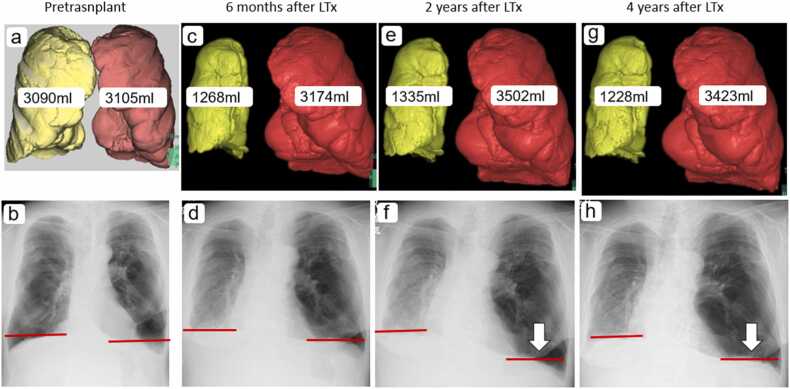
(a, b) Before lung transplantation. (c, d) Six months after lung transplantation, the hyperinflation of the transplanted right lung has improved. (e, f) Two years after lung transplantation, expansion of the remaining native left lung was observed, but there were no signs of compression on the right lung. Instead, the left diaphragm has descended. (g, h) Four years after lung transplantation, the increase in left lung expansion showed no worsening compared to the second postoperative year, and there were no significant anatomical changes to the surrounding organs since that time.

**Figure 2:**
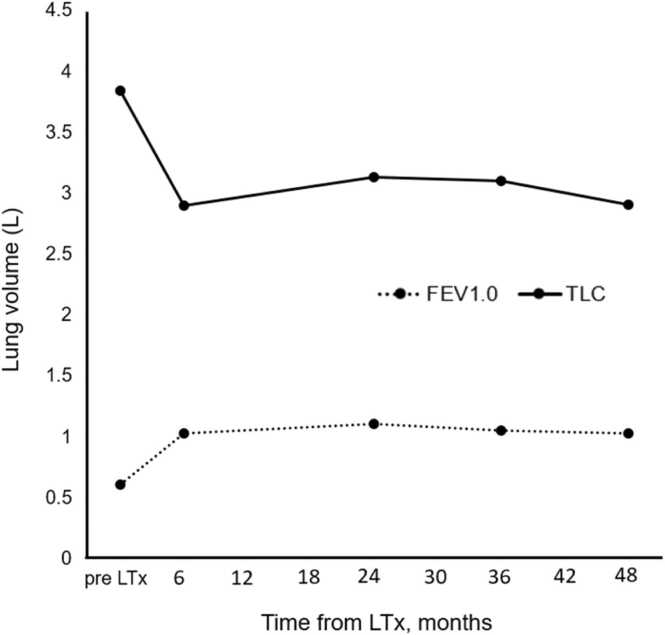
Postoperative trends of forced expiratory volume in 1 s (FEV₁) and total lung capacity (TLC) are shown. Despite the expansion of the native left lung, both FEV₁ (solid line) and TLC (dotted line) remained stable postoperatively.

Clinical research by Benvenuto et al. proposed that right single lung transplantation for COPD achieves outcomes comparable to double lung transplantation due to specific anatomical and physiological factors.^2^, ^3^ Their hypothesis suggested that hyperinflation is less severe in right single lung transplantation because the smaller native left lung and the heart's presence limit excessive hyperinflation. They also noted that left single lung transplantation carries a higher risk of postoperative infections, as the larger native right lung can compress the transplanted lung due to mediastinal shift. Additionally, the anatomical configuration of the right lung—being larger and less restricted—provides more effective ventilation and blood flow. Native lung hyperinflation (NLH) is a well-recognized complication following SLT for COPD, often resulting in compression of the graft and impaired respiratory mechanics. Motoyama et al. quantitatively evaluated NLH using three-dimensional computed tomography (3D-CT) volumetry and demonstrated its utility in detecting postoperative mediastinal shift and native lung volume changes after SLT for emphysema patients.^4^　Furthermore, Perch et al. reported a case where lung volume reduction coils were successfully used to treat symptomatic NLH following SLT, offering a minimally invasive treatment option especially for patients with collateral ventilation.^5^ These studies underscore the importance of both early radiological assessment and consideration of interventional therapies in the management of NLH post-SLT.

Building on Benvenuto's hypothesis, this case introduces a supplementary factor: the absence of the liver beneath the left diaphragm allowed the hyperinflated native left lung to expand predominantly downward, pushing the diaphragm rather than compressing the mediastinum or the transplanted right lung.^6^ The observed preservation of graft function in this case may be partially explained by the anatomical advantage of the left hemidiaphragm, which is known to exhibit greater downward excursion due to the absence of the liver. This physiological feature likely contributed to the accommodation of the hyperinflated native left lung without mediastinal shift. Notably, our use of serial CT volumetry and 3D imaging provided objective evidence of this dynamic expansion pattern, which we believe adds novelty and educational value to this report. This unique expansion pattern helped preserve the transplanted lung's function, avoiding the complications commonly associated with native lung hyperinflation in single lung transplantation.

In contrast, in left single lung transplantation, the hyperinflated native right lung often exerts pressure on the mediastinum and transplanted lung. Unlike the left lung, the right lung cannot expand downward due to the liver beneath the right diaphragm, leading to a higher likelihood of mediastinal shift, mechanical compression, impaired ventilation, and increased infection risk.

This case supports Benvenuto's hypothesis and additionally highlights that downward expansion of the native left lung—facilitated by the absence of the liver—may help preserve graft function. These anatomical features may partially explain favorable outcomes in right single lung transplantation for COPD.

One limitation of this report is that it is based on a single patient observation, which may not be generalizable. In particular, the recipient’s body habitus may influence the downward mobility of the left diaphragm. Although the present case had a BMI of 25.8 kg/m² without apparent restriction in diaphragmatic excursion, more severe overweight or obesity may potentially interfere with diaphragmatic movement, limiting the compensatory mechanism proposed in this study.

While this report provides a detailed volumetric analysis in a single case, further studies involving larger cohorts and direct comparisons between right and left single-lung transplant recipients are warranted. Longitudinal analysis of volumetric changes in both the transplanted and native lungs could offer a more comprehensive understanding of native lung hyperinflation and its long-term clinical implications in COPD.

In conclusion, the downward hyperinflation of the native left lung avoided compression of the transplanted right lung, preserving its function. This case underscores the need to consider anatomical and physiological factors, including diaphragm mobility, when evaluating outcomes of single lung transplantation for COPD.

## Declaration of Interest Statement

The authors declare that they have no known competing financial interests or personal relationships that could have appeared to influence the work reported in this paper.

## Patient consent

The authors confirm that appropriate patient consent to publish this case report was received.

## Funding

This study was funded by a JSPS KAKENHI grant [22K16570 to S.T.].
